# Superlow Power Consumption Artificial Synapses Based on WSe_2_ Quantum Dots Memristor for Neuromorphic Computing

**DOI:** 10.34133/2022/9754876

**Published:** 2022-09-13

**Authors:** Zhongrong Wang, Wei Wang, Pan Liu, Gongjie Liu, Jiahang Li, Jianhui Zhao, Zhenyu Zhou, Jingjuan Wang, Yifei Pei, Zhen Zhao, Jiaxin Li, Lei Wang, Zixuan Jian, Yichao Wang, Jianxin Guo, Xiaobing Yan

**Affiliations:** ^1^Key Laboratory of Brain-Like Neuromorphic Devices and Systems of Hebei Province, College of Electronic and Information Engineering, Hebei University, Baoding 071002, China; ^2^Department of Clinical Laboratory Medicine, Taizhou Central Hospital (Taizhou University Hospital), Taizhou 318000, China; ^3^College of Physics Science and Technology, Hebei University, Baoding 071002, China

## Abstract

As the emerging member of zero-dimension transition metal dichalcogenide, WSe_2_ quantum dots (QDs) have been applied to memristors and exhibited better resistance switching characteristics and miniaturization size. However, low power consumption and high reliability are still challenges for WSe_2_ QDs-based memristors as synaptic devices. Here, we demonstrate a high-performance, superlow power consumption memristor device with the structure of Ag/WSe_2_ QDs/La_0.3_Sr_0.7_MnO_3_/SrTiO_3_. The device displays excellent resistive switching memory behavior with a *R*_OFF_/*R*_ON_ ratio of ~5 × 10^3^, power consumption per switching as low as 0.16 nW, very low set, and reset voltage of ~0.52 V and~ -0.19 V with excellent cycling stability, good reproducibility, and decent data retention capability. The superlow power consumption characteristic of the device is further proved by the method of density functional theory calculation. In addition, the influence of pulse amplitude, duration, and interval was studied to gradually modulating the conductance of the device. The memristor has also been demonstrated to simulate different functions of artificial synapses, such as excitatory postsynaptic current, spike timing-dependent plasticity, long-term potentiation, long-term depression, and paired-pulse facilitation. Importantly, digit recognition ability based on the WSe_2_ QDs device is evaluated through a three-layer artificial neural network, and the digit recognition accuracy after 40 times of training can reach up to 94.05%. This study paves a new way for the development of memristor devices with advanced significance for future low power neuromorphic computing.

## 1. Introduction

The human brain is a sophisticated and highly efficient information processing and storage system, including approximately 10^11^ neurons, and more than 10^14^ synapse connections [1–3]. The complicated neural network can process a large amount of information at the same time with a much lower power consumption of ~20 W [4, 5]. It performs better than traditional computers on complex tasks owing to the intrinsic characteristics of the integration of storage and computing, a key to overcoming the bottleneck of the von Neumann architecture [6–8]. However, there is an urgent need for a basic unit with a simple structure to simulate biological synaptic activities to realize the intricate artificial neural network while reducing the huge demand for basic devices [3, 9–15]. Memristor, as one of the most promising technologies for constructing simulated neural networks for neuromorphic computing, has reconfigurable history-dependent resistance switching behavior and is competent to simulate the synaptic function of biological synapses [16, 17]. Although the great potential of the memristor in neuromorphic computing has been witnessed, its electrical characteristics, power consumption, linear conductance modulation, and other characteristics still need to be further improved [18–20].

In recent years, transition metal dichalcogenides (TMDs) have received extensive attention owing to their excellent electronic, optical, and mechanical properties and extensive applications [21]. Among them, WSe_2_ displays unique electrical and optical properties endowed by its high surface area and increased active edge sites urgently desired by plenty of practical applications [22, 23]. It has the advantages of high in-plane carrier mobility and electrostatic modulation of conductance and has been proven to be the first TMD material with bipolar transport characteristics, which opens up the opportunity for making high-performance nanoelectronic devices [24]. By transforming the 2D layered WSe_2_ to zero dimension (WSe_2_ quantum dots (QDs)) with a diameter of less than 10 nm, the quantum confinement and edge effects will cause additional electrical properties to be revealed [25, 26]. Due to the excellent features of WSe_2_ QDs, and the unique features of QDs-based memory, such as simple sandwiched structure, fast operation, low power consumption, and low-cost fabrication, several researchers have applied WSe_2_ QDs to the construction of memristors [21, 27]. For example, Perumalveeramalai et al. demonstrated a flexible memristor prepared by WSe_2_ QDs sandwiched between two poly(methyl methacrylate) layers with a retention time of 7 × 10^3^ s, switching endurance up to 100 cycles, desirable ON/OFF current ratio of 10^4^ [23]. However, the application of WSe_2_ QDs-based memristors in synaptic devices remains to be further investigated, and the realization and research of the synaptic plasticity in WSe_2_ QDs memristors with low power consumption and high reliability will further equip the analogue neural networks for neuromorphic computing.

In this work, the resistive switching memristor with a novel device structure of Ag/WSe_2_ QDs/La_0.3_Sr_0.7_MnO_3_ (LSMO)/SrTiO_3_ (STO) is presented, in which the Ag and WSe_2_ QDs layer, LSMO layer, and STO layer were used as top electrode, active layer, bottom electrode, and buffer layer, respectively. According to the research results of Xu et al. [28], the LSMO bottom electrode has high self-resistance compared with traditional metal bottom electrodes, which can be used as a series resistor to provide compliance current; so, the device structure can be simplified. Meanwhile, the reset process can be implemented at low current, thereby reducing the energy consumption of the device. Fabricated WSe_2_ QDs-based memristor device demonstrates excellent resistive switching characteristics with good data retention capability up to 1.5 × 10^4^ s, switching endurance up to 100 cycles, desirable *R*_OFF_/*R*_ON_ ratio of ~5 × 10^3^ with good cycling stability. Moreover, an ultra-low set voltage (*V*_set_) of ~0.52 V, reset voltage (*V*_reset_) of ~-0.19 V, and power consumption per switching of 0.16 nW are achieved, which are much lower than that of other QDs-based memristors, as illustrated in Table S1 in the Supplementary Material. In addition, the superlow power consumption characteristic of the device is further demonstrated by density functional theory calculation. Furthermore, conduction regulation can be obtained by changing pulse amplitude, duration, and interval of the pulse sequences. According to the change of conductance representing synaptic weight, various synaptic functions such as excitatory postsynaptic current (EPSC), spike timing-dependent plasticity (STDP), long-term potentiation (LTP), long-term depression (LTD), and paired-pulse facilitation (PPF) are observed to simulate the biosynaptic behavior with proper rehearsal. More importantly, digit recognition ability based on the WSe_2_ QDs device is verified according to a three-layer artificial neural network (ANN), and the digit recognition accuracy after 40 times of training can reach up to 94.05%. The fabricated Ag/WSe_2_ QDs/LSMO/STO device could be further developed and applied for constructing neural network for future neuromorphic computing architecture.

## 2. Results

**Figure 1:**
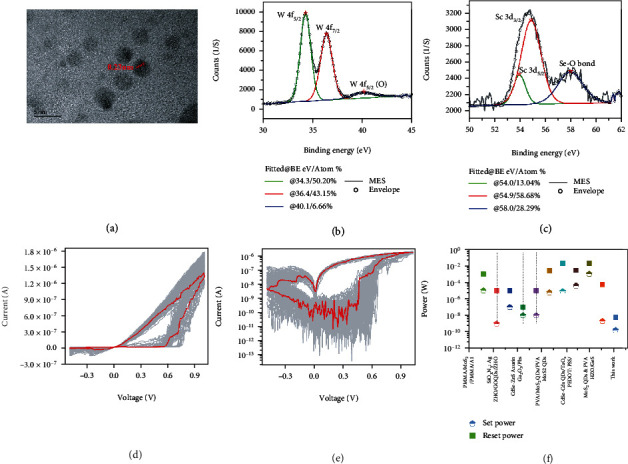
(a) HR-TEM image of WSe_2_ QDs. (b) and (c) are XPS analysis results of WSe_2_ QDs: (b) W 4f core spectra and (c) Se 3d core spectra. (d) *I*-*V* curves of Ag/WSe_2_ QDs/LSMO/STO memristor clearly display resistive switching characteristics. (e) The logarithm form of (d). (f) Comparison of the power consumption of the device with the values of other QDs-based memristors.

To observe the morphology of WSe_2_ QDs, high-resolution transmission electron microscope (HR-TEM) image was acquired and shown in Figure 1(a). The dark spots in Figure 1(a) exhibit that WSe_2_ QDs have clear boundaries and circular properties within a size range from 1.6 nm to 3.44 nm. The clearly visible lattice fringe spacing of the quantum dots is 0.23 nm, which is consistent with the literature [23]. In addition, the cross-sectional scanning electron microscope (SEM) image of the WSe_2_ QDs/LSMO/STO device was achieved, as illustrated in Figure S1, which shows that the thickness of the WSe_2_ QDs active layer is approximately 97 nm. Furthermore, to verify the successful deposition while identifying the chemical composition and states of LSMO bottom electrode and WSe_2_ QDs active layer, the X-ray photoelectron spectroscopy (XPS) measurements were executed. The XPS detection results of main elements (C, O, La, Sr, Mn, O, Se, and W) of LSMO/STO and WSe_2_ QDs/LSMO/STO were analyzed by CasaXPS (Version 2.3.13Dev29). Figure S2 in the Supplementary Material shows the XPS analysis result of the wide spectra and the core spectra of La 3d, Sr 3d, Mn 2p, and O 1 s of LSMO/STO, which clearly show the successfully formation of the LSMO bottom electrode film. The XPS wide spectra of WSe_2_ QDs/LSMO/STO are exhibited in Figure S3 in the Supplementary Material. Figure 1(b) demonstrates the core spectra of W 4 f. The peaks located at 34.3 and 36.4 eV represent W 4f_5/2_ and W 4f_7/2_, respectively, proving the appearance of the oxidation state of W^4+^ on the surface of the WSe_2_ QDs film [29, 30]. The peak located at 40.1 eV can be attributed to W 4f_5/2_ for W^6+^ (WO_3_), which may be due to surface oxidation [31]. Figure 1(c) shows the core spectra of Se 3d. The peaks located at 54.0 and 54.9 eV represent Se 3d_5/2_ and Se 3d_3/2_, respectively [32]. Through the above XPS analysis of WSe_2_ QDs/LSMO/STO, the presence of W and Se in WSe_2_ QDs is clearly characterized. The calculated chemical stoichiometric ratio of W and Se is about 1 : 1.34, demonstrating that there are selenium vacancies in our prepared WSe_2_ QDs film. Figure 1(d) depicts the current-voltage (*I-V*) curves over 100 cycles of the Ag/WSe_2_ QDs/LSMO/STO device in the voltage sweep mode of 0 V⟶1 V⟶0 V⟶−0.5 V⟶0 V. The corresponding logarithmic form of *I-V* curves is illustrated in Figure 1(e). The device indicates typical bipolar resistance switching behavior, with the transition of high-resistance state (HRS) and low-resistance state (LRS). As the positive scanning voltage increases and reaches *V*_set_~0.52 V, the memristor changes from HRS to LRS with a steeply incremental current from ~0.2 nA to 1 *μ*A when applying a sweep voltage of 0 V ~ +1 V. During the reverse scanning from +1 V to -0.5 V, the memristor realizes the conversion from LRS to HRS under a *V*_reset_ of ~-0.19 V. It is interesting to note that the set and reset power consumption of the Ag/WSe_2_ QDs/LSMO/STO device are as low as ~0.16 nW (*P*_set_ = *V*_set_ × *I*_set_) and~6 nW (*P*_reset_ = *V*_reset_ × *I*_reset_), respectively, which are much lower than many reported QDs-based memristors [21, 33–41], as illustrated in Figure 1(f). Over 100 cycles of the *I-V* sweeps, the device displayed rather robust *I-V* curves and did not degrade, showing good endurance. In addition, a set of control experiments were conducted to verify the resistance switching behavior of WSe_2_ QDs active layer. The *I-V* curves of the Ag/LSMO/STO device without spin-coated WSe_2_ QDs layer (Figure S4 in the Supplementary Material) under the same experimental conditions show linear relationships of the voltage and current when the applied voltage is 1 ~ 5 V, which suggests that the WSe_2_ QDs layer is the main reason for the resistance switching characteristics of Ag/WSe_2_ QDs/LSMO/STO device.

**Figure 2:**
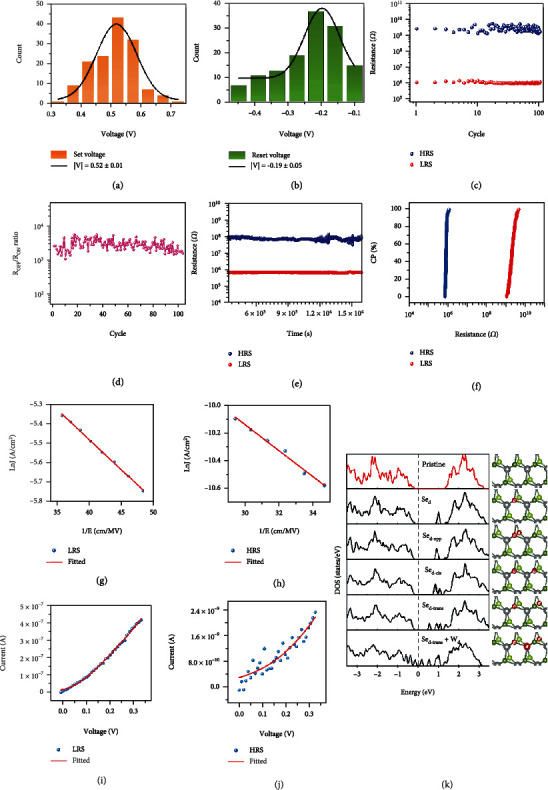
(a, b) Distribution histogram and Gaussian fitted curves of set and reset voltage. (c) Statistics of high and low resistance over 100 cycles. The read voltage is 0.2 V. (d) The ratios of *R*_OFF_/*R*_ON_ of Ag/WSe_2_ QDs/LSMO/STO device. (e) Retention data at HRS and LRS of the device in the room temperature. The read voltage is 0.5 V. (f) The cumulative probability plot of the HRS and LRS. (g) and (h) are the linear fitted curves of LRS and HRS by ln(*J*) ∝ 1/*E*, demonstrating the trap-assisted tunneling (TAT) conduction mechanism. (i) and (j) are the fitted curves of LRS and HRS by formulas (1)–(3). (k) The density of states of pristine WSe_2_ and the five defect models. The corresponding crystal structures are also shown. The dark green, light green, and gray balls represent upper layer Se atoms, lower layer Se atoms, and W atoms, respectively; “*d*” represents defect sites.

To further study the uniformity of the device, the distribution of switching voltages of the device is analyzed and shown in Figures 2(a) and 2(b). The histogram statistics of the *V*_set_ and *V*_reset_ distributions over 100 cycles were performed by Gaussian fitting analysis (the black lines are the fitted curves). The *V*_set_ and *V*_reset_ of the device were confined in the range of 0.30 to 0.75 V and -0.15 to -0.49 V, respectively. The corresponding Gaussian fitted values of the *V*_set_ and *V*_reset_ were (0.52 ± 0.01) V and (−0.19 ± 0.05) V, respectively. The distribution of the switching voltages of the device was concentrated and less diffuse, which is conducive to the realization of accurately control and read of set and reset process, as well as the future practical application of Ag/WSe_2_ QDs/LSMO/STO device. The low threshold voltage is very advantageous to reduce the power consumption of the memristor device. The distribution of HRS, LRS, and the *R*_OFF_/*R*_ON_ ratios of the device is illustrated in Figures 2(c) and 2(d). The low and high resistance values are distributed on the order of 10^6^ and 10^9^, respectively. The device can maintain 10^2^ switching cycles, indicating the excellent endurance ability, and the ratios of *R*_OFF_/*R*_ON_ between the HRS and LRS can up to ~5 × 10^3^. Moreover, both HRS and LRS displayed long retentions of 10^4^ s at the reading voltage of 0.5 V without being reduced, showing excellent stability (Figure 2(e)). The cumulative probability of HRS and LRS of the device is displayed in Figure 2(f), demonstrating the distinguishable HRS and LRS with the *R*_OFF_/*R*_ON_ ratio of ~5 × 10^3^.

To further understand the conduction mechanism of Ag/WSe_2_ QDs/LSMO/STO device, the switching characteristics were studied throughout the whole testing process. The analysis and fitting results are illustrated in Figures 2(g)–2(j). LRS and HRS of the *I-V* curves are fitted by the linear function ln (*J*) ∝ 1/*E*, respectively (Figures 2(g) and 2(h)), and the results indicate that the conductive characteristic of the device is in accordance with the TAT conduction mechanism [42]. HRS and LRS of the *I-V* curves can be fitted with formulas (1)–(3). Based on the conductance theory of TAT conduction mechanism, the tunneling current (*I*) can be expressed as [19, 43]

(1) $$\begin{array}{c} I=N\times q\times v \end{array}$$

Here, *N* represents the total number of the closest traps that conduce to the conduction, and the transition rate *v* can be expressed as

(2) $$\begin{array}{c} v=v_{0}\times f\times P \end{array}$$

*v*_0_ represents the frequency factor, and the Fermi-Dirac distribution of electrons in the electrode can be calculated as *f* = 1/[1 + exp(*E*_*b*_ − *E*_*t*_ + *F* × *d*)/*kT*)]. *E*_*b*_ represents the height of the barrier between the electrode and the conduction band, and *k* and *T* are Boltzmann constant and room temperature, respectively. The transmission probability *P* can be defined as

(3) $$\begin{array}{c} P=\exp\left\{-\frac{4}{3\hbar qF}\sqrt{2m_{c}}\left[{E_{t}}^{3/2}-{\left(E_{t}-F\times d\right)}^{3/2}\right]\right\} \end{array}$$

*ħ* and *q* represent the reduced Planck's constant and electronic charge quantity, respectively. *F*, *d*, and *E*_*t*_ represent the electric field intensity, tunneling distance, and defect trap energy lower than the conduction band, respectively [44]. By fitting with formulas (1)–(3), two parameters can be received from the fitting results in Figures 2(i) and 2(j): the tunneling distance *d* and the trap energy *E*_*t*_. Figures 2(i) and 2(j) exhibit the fitting results of the *I-V* curves obtained by adjusting *d* and *E*_*t*_, where *N* is regarded as a constant [45]. From the fitting results of LRS (Figure 2(i)), *E*_*t*_ and *d* are 1.32 eV and 0.4 nm, respectively. In HRS (Figure 2(j)), *E*_*t*_ and *d* are slightly increased to 1.44 eV and 0.41 nm, respectively. The obtained results illustrate that HRS has deeper defect energy level traps and larger tunneling distances. In the TAT model, the movement of electrons is realized with the aid of defects [42]. Therefore, the lower trap energy *E*_*t*_ and distance *d* in LRS are beneficial to carrier transport.

The chemical stoichiometric ratio of *W* and Se obtained by XPS characterization indicates that there are enormous number of Se-site defects (Se*_d_*) in our prepared WSe_2_QDs films, which is consistent with the reports that chalcogen defects are generally supposed to be the most common intrinsic defects in TMDs [46, 47]. Therefore, we investigated the defect formation energies and defect electronic structures of several defect models in WSe_2_, including one Se*_d_* and the composite defects. For the composite defects, two Se*_d_*, as well as two Se*_d_* containing one *W*-site defect (*W*_*d*_), are considered, whereas the above composite defects with two Se*_d_* may be arranged in opposite (*opp*), *cis*, or *trans* configurations. The defect formation energies for one Se*_d_* and the composite defect models are listed in Table S2 in the Supplementary Material. Figure 2(f) illustrates the calculated density of states (DOS) diagrams of pristine WSe_2_ and the five most preferred defect models, *i.e.*, the structure with one Se*_d_*, the opposite, *cis*, and *trans* configurations of two Se*_d_* (Se_*d-*opp_, Se_*d-*cis_, and Se_*d*-trans_), and the *trans* configuration of two Se*_d_* containing one *W*-site defect (Se_*d-*trans_ + *W*_*d*_). The computational details are shown in the Supplementary Material. As shown in Figure 2(f), the electronic structure of the pristine WSe_2_ shows a band gap of about 1.6 eV, which is consistent with the previous report [48]. The presence of a Se*_d_* and composite defects prefers to lead to the generation of defect states in the band gap of WSe_2_. A single Se*_d_* can create a single defect state 0.28 eV below the conduction band. In addition, the case of the spatial configuration with 2Se*_d_* proves to be extremely meaningful. Unlike a single Se*_d_*, the presence of a second Se*_d_* results in the change of defect states and band energy of the opposite configuration but creates new and different defect states for the *trans* configurations. However, due to the composite defects of two Se*_d_* and *W*_*d*_ in the *trans* configurations, the defect state system distributed throughout the whole band gap is generated. The above analysis proves that the defect states formed by one Se*_d_* and composite defects are at deep energy levels with localized characteristics; therefore, current leakage is not prone to occur, which further explains and demonstrates the superlow consumption characteristic of the Ag/WSe_2_ QDs/LSMO/STO device [19].

**Figure 3:**
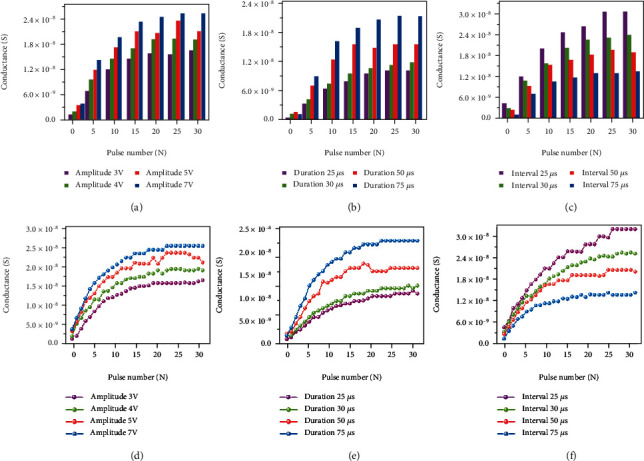
The conductance of the device for 30 pulse cycles was measured under different pulse (a) amplitudes, (b) durations, and (c) intervals. The device conductance was measured under a train of positive pulses: (d) the pulse duration and interval are both 50 *μ*s and different pulse amplitudes. (e) The pulse amplitude and interval are 4 V and 50 *μ*s, respectively, and different pulse durations. (f) The pulse amplitude and duration are 4 V and 50 *μ*s, respectively, and different pulse intervals.

Similar to biological synapses, the conductance of our WSe_2_ QDs-based device can not only be modulated by the pulse amplitude and duration but also by the pulse interval, which proves the synaptic plasticity of our device. To further investigate the conductance modulation properties of Ag/WSe_2_ QDs/LSMO/STO device, a series of positive pulse sequences were introduced to the device. The controllability of the conductance modulation was investigated by changing the amplitude, duration, and interval of the applied pulse. The conductance of the device was recorded instantly after the excitation was applied, and the serials of pulses were expressed by different colors.  Figure 3(a) indicates that the conductance and the amplitude of the device are positively correlated under the condition of the same number of pulses; that is, the conductance increases with the increasing pulse amplitude (the pulse interval and duration are both fixed at 50 *μ*s).  Figure 3(b) indicates that the conductance increases with the increasing pulse duration (the pulse amplitude is 4 V, and the pulse interval is 50 *μ*s).  Figure 3(c) illustrates that the conductance and the pulse interval are negatively correlated; in other words, the conductance decreases with the increasing pulse interval (the pulse amplitude is 4 V, and the pulse duration is 50 *μ*s). The effect of the pulse amplitude on the variation of the conductance is illustrated in Figure 3(d) under the constant pulse interval and duration (the pulse interval and duration are both fixed at 50 *μ*s): a higher amplitude will result in an increased rate of rise in conductance and reach the saturated conductance value. The effect of the pulse duration on the variation of the conductance is illustrated in Figure 3(e) with a constant amplitude and interval (i.e., 4 V and 50 *μ*s). The results suggest that the rate of conductance increases as the pulse duration increases. The influence of the pulse interval on the variation of the conductance is shown in Figure 3(f) with a constant amplitude and duration (i.e., 4 V and 50 *μ*s), but the opposite result from Figure 3(e) is observed: the rate of conductance decreases with the increase of the pulse interval. In general, the conductance of the device can be finely modulated by the pulse number, amplitude, duration, and interval, which is conducive to the simulation of biological synaptic functions.

**Figure 4:**
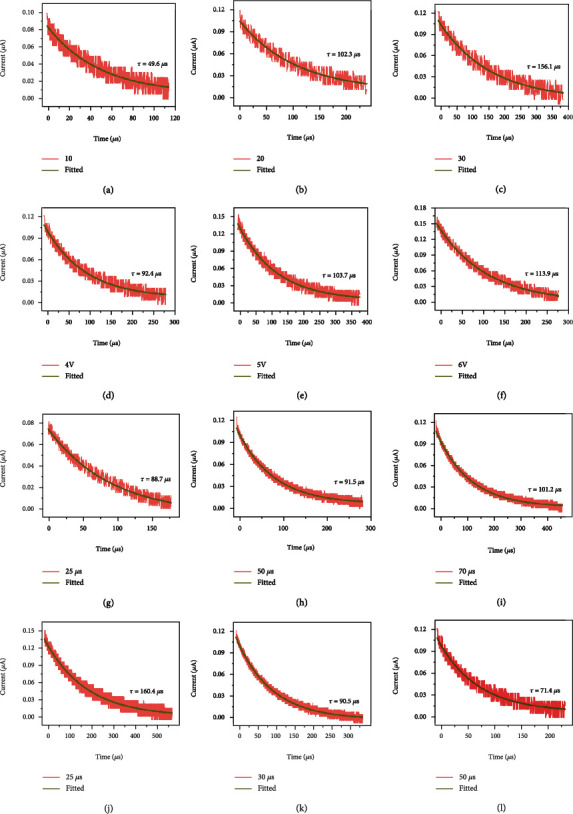
(a)–(c) Comparison of EPSC measurement response (orange curves) and fitted curves (green curves) under the condition of different pulse numbers. (d)–(f) Comparison of EPSC measurement response (orange curves) and fitted curves (green curves) under different square wave amplitudes. (g)–(i) Comparison of EPSC measurement response (orange curves) and fitted curves (green curves) under different pulse durations. (j)–(l) Comparison of EPSC measurement response (orange curves) and fitted curves (green curves) at different intervals.

The forgetting curve of human memory is closely related to the approach of learning information. The “learning approach” (that is, the stimulus conditions) changes with the stimulation amplitude, duration, and interval [49]. EPSC means that the action signals and potentials of presynaptic neurons are transmitted to postsynaptic neurons through synapses under the action of an external excitation source.  Figure 4 shows that when pulses of different numbers, amplitudes, durations, and intervals were applied to the device, the tail wave after the last pulse of each stimulation was measured and recorded [50]. After removing the applied square wave voltage, the synaptic weight would decay spontaneously in the absence of external inputs [51].

The correspondence between the forgetting behavior of the Ag/WSe_2_ QDs/LSMO/STO device and the short-term plasticity (STP) of human neurons was investigated through an exponential decay equation describing the STP relaxation process:

(4) $$\begin{array}{c} M\left(t\right)=M_{e}+\left(M_{0}-M_{e}\right)\exp\left(\frac{-t}{\tau }\right), \end{array}$$

where *M*_0_ and *M*_*e*_ represent the initial and stable memory state, respectively, and *τ* represents the relaxation time constant. A larger *τ* value indicates a slower forgetting rate [50, 51]. Figures 4(a)–4(c) depict EPSC response results under different stimulation times. The fitted values of *τ* are 49.6 *μ*s (Figure 4(a)), 102.3 *μ*s (Figure 4(b)), and 156.1 *μ*s (Figure 4(c)). If the applied number of pulses is larger, the stimulation time is longer, and the value of *τ* is larger, i.e., the forgetting is slower. Figures 4(d)–4(f) depict the response results corresponding to different amplitudes, where the fitted *τ* values are 92.4 *μ*s (Figure 4(d)), 103.7 *μ*s (Figure 4(e)), and 113.9 *μ*s (Figure 4(f)). The results illustrate that the greater the amplitude, the greater the value of *τ*, i.e., the slower the forgetting. Figures 4(g)–4(i) show the response results of different durations, with different *τ* values of 88.7 *μ*s (Figure 4(g)), 91.5 *μ*s (Figure 4(h)), and 101.2 *μ*s (Figure 4(i)). The results show that a larger pulse duration will result in a larger *τ* value, i.e., a slower forgetting rate. Figures 4(j)–4(l) are the response results with respect to different intervals, and the fitted *τ* values are 160.4 *μ*s (Figure 4(j)), 90.5 *μ*s (Figure 4(k)), and 71.4 *μ*s (Figure 4(l)), respectively. The results show that a smaller interval will result in a larger *τ* value, i.e., a slower forgetting rate. The above analysis suggests that our device can realize EPSC simulation commendably.

**Figure 5:**
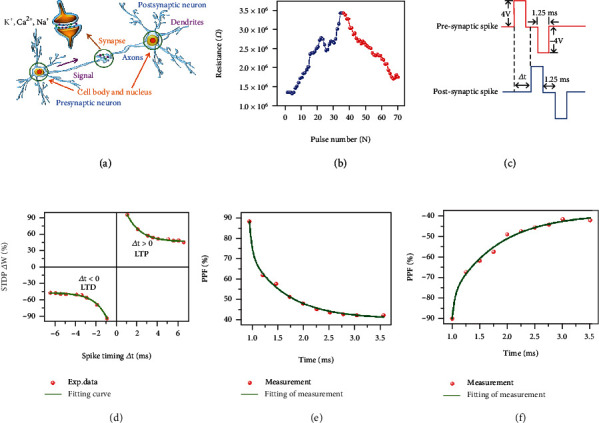
Simulation of the characteristics of STDP and PPF in biological synapses. (a) Schematic illustration of the structure of biological synapses. (b) The relationship between the pulse number and the resistance of the device. Applying a negative/positive pulse to the device will cause a decrease/increase in resistance, which represents the modulation of synaptic weight owing to enhancing or suppressing the pulses. (c) Schematic diagram of the pulse waveforms applied to the device for STDP simulation. (d) Measured STDP characteristics of Ag/WSe_2_ QDs/LSMO/STO device, the green lines are the curves fitted by Equation (5). (e, f) Measured PPF characteristics of Ag/WSe_2_ QDs/LSMO/STO device, (e) and (f) are the test results after applying positive and negative voltage pulses, respectively.

STDP is one of the most significant biological features in the Hebbian learning rules for learning and memory, which can regulate the connection strength between human brain neurons [52, 53].  Figure 5(a) is a schematic illustration of a biological synapse, which is the connection between two neurons. The structure of Ag/WSe_2_ QDs/LSMO/STO memristor device is similar to a typical nerve synapse. The top electrode (Ag) is considered as the presynaptic membrane, while the bottom electrode (LSMO) is considered as the postsynaptic membrane. Previous studies have demonstrated that metal ions such as Ag^+^ and Cu^2+^ can migrate under the application of electric field and form conductive filaments, which is able to simulate the weight change of biological synapses caused by the release of Ca^2+^ or Na^+^ from preneurons [3]. For devices based on Ag/WSe_2_ QDs/LSMO/STO structure, the change of device resistance is studied when the driving voltage pulse sequence is applied. The setting mode of programming voltage is as below: the negative voltage pulse part is −7⟶−0.2 V, the voltage change step is -0.2 V; the positive voltage pulse part is 0.2⟶7 V, and the voltage change step is 0.2 V. The duration and interval of each pulse are both 41.5 *μ*s, and the obtained resistance change of the device is illustrated in Figure 5(b). In the negative voltage pulse part (blue), the absolute voltage value is increasing, and the resistance of the device increases with the decrease of negative voltage (depression). In the positive voltage pulse part (red), the absolute voltage value is increasing, and the resistance of the device decreases with the increase of positive voltage (potentiation). Therefore, the regulation of the weight of biological synapses (i.e., the variations of connection strength between biological synapses) can be simulated by the change of memristor resistance. STDP adjusts the synaptic weight by changing the interval from presynaptic to postsynaptic peaks (Δ*t*). If the prestimulation time of the neuron is earlier than the poststimulation time of the neuron (i.e., Δ*t* > 0), an increase in the postsynaptic current will caused. The phenomenon indicates that the stimulation signal of presynaptic neuron can be conducive to promote the producing of postsynaptic neuron stimulation signal, and the synapse weight increases more as |Δ*t*| decreases. On the contrary, if the presynaptic stimulation time is later than the postsynaptic stimulation time (i.e., Δ*t* < 0), the postsynaptic current will be inhibited. This indicates that the stimulation signal of presynaptic neurons plays an inhibitory role in the generation of postsynaptic neuron stimulation signals, and the synaptic weight decreases more as |Δ*t*| decreases. The STDP rule what we generally referred to occurs in the time window between excitement and excitement. When the action potential of presynaptic neurons is earlier than that of postsynaptic neurons, the weight of synaptic will increase, signifying LTP [34, 54–57]. On the contrary, when the action potential of presynaptic neurons is later than that of postsynaptic neurons, the weight of synaptic will decrease, signifying LTD [58]. This is called the anti-Hebbian learning rule. Following the above rules and definitions, we designed presynaptic and postsynaptic spike waveforms (as shown in Figure 5(c)) to stimulate Ag/WSe_2_ QDs/LSMO/STO synapses, and the results (as shown in Figure 5(d)) demonstrate our device can implement this rule well. The fitted curve in Figure 5(d) is expressed by Equation (5):

(5) $$\begin{array}{c} \Delta W=Ae^{-\Delta t/\tau }+\Delta W_{0} \end{array}$$

Here, *A* is the scale factor of the STDP function, *τ* is the time constant [59, 60], and *W*_0_ is a constant which represents the nonassociative part of the synaptic change.

PPF is a typical physiological phenomenon in which the synaptic weight of biological synapses is increased in a short time during the continuous release of calcium ions at the presynaptic end owing to the presynaptic influx of ions. In a pair of presynaptic stimuli, when the second stimulus is triggered within a short time interval, the post-synaptic response of the second stimulus will be greater than that of the first stimulus, resulting in synaptic weights [61, 62]. In order to prove the PPF phenomenon in our device, a pulse with a pulse duration of 1.25 ms and a voltage amplitude of ±1 V was applied to Ag top electrodes. The correlation between synaptic weight and pulse time interval are shown in Figures 5(e) and 5(f). The pulse waveforms applied to the device for PPF simulation are illustrated in Figure S5 in the Supplementary Material. The ratios of PPF are expressed by [37]:

(6) $$\begin{array}{c} \text{PPF}=\frac{\left(G_{2}-G_{1}\right)}{G_{1}}\times 100\%=C_{1}\exp\left(\frac{-t}{\tau_{1}}\right)+C_{2}\exp\left(\frac{-t}{\tau_{2}}\right) \end{array}$$

Here, *G*_1_ and *G*_2_ are the conductance values after the action of the previous and subsequent pulses, respectively, and *τ*_1_ and *τ*_2_ are the fitted time constants, corresponding to the fast and slow decaying components, respectively [18]. For a positive voltage pulse, the fitted *τ*_1_ and *τ*_2_ are 48 *μ*s and 700 *μ*s, respectively (Figure 5(e)), while for a negative voltage pulse, the fitted *τ*_1_ and *τ*_2_ are 48 *μ*s and 855 *μ*s, respectively (Figure 5(f)). Our results indicate that as the pulse interval was decreased, the memory effect of the prespiking pulse on subsequent pulses was improved, which is excellently consistent with biological synapses.

**Figure 6:**
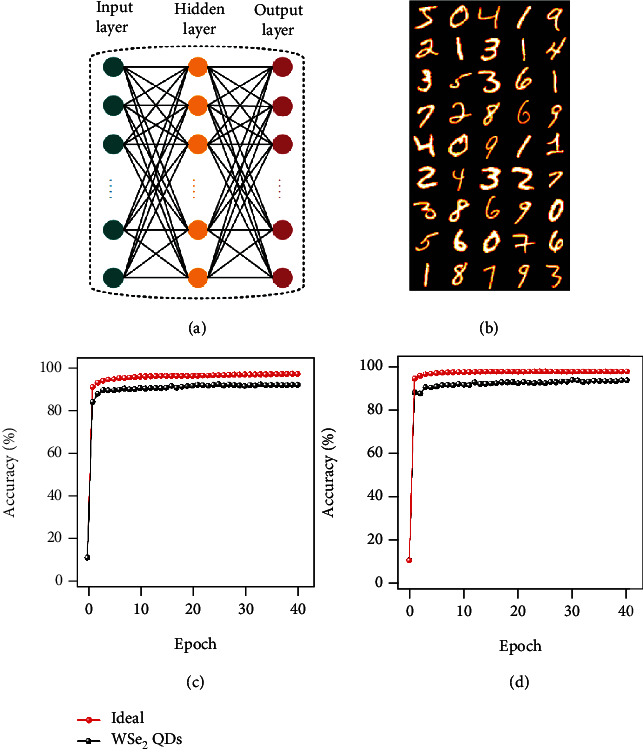
Simulation of neural network based on WSe_2_ QDs device. (a) A three-layer neural network structure is shown. It contains input layer, hidden layer and output layer. (b) Image representation of some handwritten digits in the MNIST dataset. (c) The comparison between the recognition accuracy of the optical recognition dataset of handwritten digits in the neural network simulation and the ideal case. After 40 training sessions, the ideal case recognition accuracy reaches 96.71%, and the device-based recognition accuracy reaches 91.59%. (d) Regarding the comparison of the recognition accuracy of the MNIST dataset in the neural network simulation with the ideal case, after 40 training sessions, the ideal case recognition accuracy reaches 98.19%, and the device-based recognition accuracy reaches 94.05%.

To better evaluate the application of the Ag/WSe_2_ QDs/LSMO/STO device in neuromorphic computing, we built a three-layer ANN to simulate the performance of the WSe_2_ QDs-based memristor, including the input layer, hidden layer, and output layer in the network, as illustrated in Figure 6(a). Here, two datasets are used for evaluation, small images (8 × 8 pixels) of hand-written digits from the “Optical Recognition of Handwritten Digits” (ORHD) dataset [63] and large images (28 × 28 pixels) of hand-written digits from the “Modified National Institute of Standards and Technology” (MNIST) dataset [64], and the representative images of the MNIST dataset are illustrated in Figure 6(b). In the process of neural network simulation based on the WSe_2_ QDs device, the weights between the neurons will be mapped to the intersection of the horizontal bar and the vertical bar in the crossbar based on the WSe_2_ QDs device (Figure S6 in the Supplementary Material) [65]. A crossbar, considered part of the “neural core,” is used to perform vector-matrix multiplication and outer product update operations (Figure S7 in the Supplementary Material). The detailed simulation process is shown in the Supplementary Material. After 40 times of training in ANN, the recognition accuracy of WSe_2_ QDs-based device in recognizing small images reaches 91.59%, and the ideal performance of floating-point-based neural networks [66] is 96.71%, which represents the theoretical limit of the simulator, as illustrated in Figure 6(c). The recognition accuracy of WSe_2_ QDs-based device reaches 94.05% in recognizing large images, and the ideal performance of the floating-point-based neural network reaches 98.19%, as illustrated in Figure 6(d). Compared to the work of Ge et al. [66], the image recognition accuracy of our devices for large images is improved by 3.05%. The above results fully demonstrate that the WSe_2_ QDs-based device is very suitable for neuromorphic computing and provides new ideas for the further development of neuromorphic computing.

## 3. Discussion

In conclusion, we have presented a high-performance and superlow power consumption memristor device with the structure of Ag/WSe_2_ QDs/LSMO/STO. The device exhibits excellent resistive switching characteristics with stable memory performance, with decent *R*_OFF_/*R*_ON_ ratio up to 5 × 10^3^, superlow consumption of 0.16 nW, set and reset voltages as low as ~0.52 V and~ -0.19 V, and reliable repeatability. The movement of electrons assisted by defects obtained by the TAT model is responsible for the resistive switching behavior of the device. Meanwhile, density functional theory calculations demonstrate that the defect states formed by Se*_d_* and W_*d*_ are at deep energy levels; so, current leakage does not easily occur, which further explain and prove the low power consumption characteristic of the device. Moreover, conduction regulation can be achieved by changing the external conditions, such as pulse amplitude, duration, and interval. And biological synaptic characteristics including EPSC, STDP, LTP, LTD, and PPF were successively proved. The recognition accuracy of digit images obtained by a three-layer ANN can reach up to 94.05%. This work demonstrates the Ag/WSe_2_ QDs/LSMO/STO memristor device holds great potential for application in low power consumption neuromorphic computing system.

## 4. Materials and Methods

### 4.1. Fabrication of the WSe_2_ QDs Suspension

WSe_2_ QDs were prepared based on the method reported in the reference [25]. First, WSe_2_ powder was dispersed in N-methyl-2-pyrrolidone (NMP) to prepare a mortar with a concentration of 50 mg/mL. After dilution treatment in a glass vial containing 3 mL NMP and grinding for 30 min, the suspension was sonicated in an ice bath with a power of 260 W for 4 h. After regrinding for 30 minutes, the suspension was diluted to 15 mL and then resonication in an ice bath for another 4 h with a power of 260 W. After that, the resulting suspension was centrifuged at 8000 rpm for 20 minutes. After two cycles of centrifugation, the supernatant was collected and finally filtered with a 250 nm Teflon filter. In order to prevent WSe_2_ from being oxidized, the entire ultrasonic treatment was carried out in a nitrogen atmosphere.

### 4.2. Fabrication of the Device

The sandwich structure device was fabricated by a combination of pulsed laser deposition (PLD), spin coating, and magnetron sputtering technology. First, the P-type Si substrate with a 1 *μ*m-thick SiO_2_ layer was cleaned with acetone, ethanol, and deionized water (DIW), respectively and then immersed in a mixture of hydrofluoric acid and DIW (1 : 3) to remove the silicon dioxide. Next, the STO buffer layer was deposited on the Si substrate by PLD under a growth temperature at 750°C, an oxygen pressure at 7.5 mTorr, and a laser repetition frequency of 5 Hz for 15 min. Then, the LSMO bottom electrode was deposited using a laser with an energy density of 350 mJ/cm^2^ and repetition frequency of 2 Hz, while maintaining the growth temperature at 750°C and oxygen pressure at 200 mTorr for 30 min. Afterward, the WSe_2_ QDs active layers were formed by spin-coating on the LSMO bottom electrode at a coating speed of 600 rpm for 60 s. Finally, an Ag top electrode film with a thickness of 60 nm and a diameter of 90 *μ*m was fabricated by direct current magnetron sputtering technology under a pressure of 3 Pa and an Argon flow rate of 25 sccm.

### 4.3. Characterizations

HR-TEM (JEM-2100HR) was applied to identify the quality of the WSe_2_ QDs. SEM (FEI Nova Nano SEM450) was utilized to identify the thickness of the WSe_2_ QDs active layer. XPS (Thermo Fischer ESCALAB Xi+) was utilized to analyze the chemical configurations of the WSe_2_ QDs using a 12.5 kV monochromatic Al K*α* source. The C 1 s peak at 284.8 eV was used for charge calibration of all the binding energies. The electrical characterization experiments of Ag/WSe_2_ QDs/LSMO/STO device, including the direct current *I-V* curves and pulse measurements, were determined at atmospheric pressure in air ambient using a Keithley 2400 digital source meter. A function/arbitrary waveform generator (RIGOL DG5102) was applied to conductance regulation experiment and tests EPSC, STDP, LTP, LTD, and PPF. An oscilloscope (RIGOL DS4022) was utilized to capture the waveforms throughout the pulse measurements. During all the electrical characterization experiments, the voltage bias was applied to the Ag electrode while the LSMO electrode and Si substrate were grounded all the time.

### 4.4. The Method Used in Digit Recognition Ability

The neural network simulations based on WSe_2_ QDs memristors were performed in Python software. The Cross Sim simulator was used for supervised learning of the ORHD dataset and MNIST dataset. There are a total of 5620 8 × 8 pixel pictures of written digits in the ORHD dataset, of which 3823 are used for training and 1797 are used for testing. There are a total of 70,000 pictures of handwritten digits in the MNIST dataset, of which 60,000 are used for training and 10,000 are used for testing. During the simulations on both datasets, a learning rate of 0.1 was used.

## Data Availability

All data needed to evaluate the conclusions in the paper are present in the paper and the Supplementary Material. Additional data related to this paper may be requested from the authors.

## Supplementary Materials

Supplementary Materials Supplementary Materials Figures and figure captions: Figure S1: the cross-sectional SEM image of the WSe_2_ QDs/LSMO/STO device. Figure S2: XPS analysis result of the LSMO/STO device. Figure S3: XPS wide spectra of the WSe_2_ QDs/LSMO/STO device. Figure S4: the *I-V* curves of the Ag/LSMO/STO device without spin-coated WSe_2_ QDs layer. Figure S5: schematic diagram of the pulse waveforms applied to the device for PPF simulation. Figure S6: the schematic diagram of the crossbar based on the WSe_2_ QDs device. Figure S7: the neural core. Figure S8: schematic diagram of digital image input. Tables and table captions: Table S1: key parameters compared with other QDs-based memristors. Table S2: the defect formation energies of defect configurations for WSe_2_. Table S3: D/A and A/D attributes.
