# Supplementary material for: Superlow Power Consumption Artificial Synapses Based on WSe2 Quantum Dots Memristor for Neuromorphic Computing
Source: Research (Wash D C). 2022 Sep 13;2022:9754876. doi: 10.34133/2022/9754876 (PMC9513833; doi:10.34133/2022/9754876)
Supplement: Supplementary Materials — Figures and figure captions: Figure S1: the cross-sectional SEM image of the WSe2 QDs/LSMO/STO device. Figure S2: XPS analysis result of the LSMO/STO device. Figure S3: XPS wide spectra of the WSe2 QDs/LSMO/STO device. Figure S4: the I-V curves of the Ag/LSMO/STO device without spin-coated WSe2 QDs layer. Figure S5: schematic diagram of the pulse waveforms applied to the device for PPF simulation. Figure S6: the schematic diagram of the crossbar based on the WSe2 QDs device. Figure S7: the neural core. Figure S8: schematic diagram of digital image input. Tables and table captions: Table S1: key parameters compared with other QDs-based memristors. Table S2: the defect formation energies of defect configurations for WSe2. Table S3: D/A and A/D attributes. [file 9754876.f1.zip › Revised Supplementary Material (Clean Version).docx]

**Supplementary Material**

**Super-low Power Consumption Artificial Synapses Based on** **WSe_2_** **Quantum Dots Memristor for Neuromorphic Computing**

Zhongrong Wang^1^†, Wei Wang^1^†, Pan Liu^1^, Gongjie Liu^1^, Jiahang Li^1^, Jianhui Zhao^1^, Zhenyu Zhou^1^, Jingjuan Wang^1^, Yifei Pei^1^, Zhen Zhao^1^, Jiaxin Li^1^, Lei Wang^1^, Zixuan Jian^1^, Yichao Wang^2^, Jianxin Guo^3^, and Xiaobing Yan^1,4^*

1 Key Laboratory of Brain-Like Neuromorphic Devices and Systems of Hebei Province, College of Electron and Information Engineering, Hebei University, Baoding, 071002, China

2 Department of Clinical Laboratory Medicine, Taizhou Central Hospital (Taizhou University Hospital), Taizhou, 318000, China

3 College of Physics Science and Technology, Hebei University, Baoding, 071002 China

4 Department of Materials Science and Engineering National University of Singapore, Singapore, 117576, Singapore

Correspondence should be addressed to Xiaobing Yan; [yanxiaobing@ime.ac.cn](mailto:yanxiaobing@ime.ac.cn)

†These coauthors contributed equally to this work

***Table S1. Key parameters compared with other QDs based memristors.***

| **Device Structure** | **V_set_/V** | **V_reset_/V** | **P_set_** | **P_reset_** | **Ref.** |
| --- | --- | --- | --- | --- | --- |
| Ag/WSe_2_ QDs/LSMO/STO | 0.52 | -0.19 | 0.16 nW | 6 nW | This work |
| Al/PMMA/MoS_2_/PMMA/Al/PET | 4.8 | -4.9 | 10 μW | 1 mW | [1] |
| Pt/SiO_x_N_y_: Ag/Pt | 0.25~0.35 | 0.01~0.12(thresold) | 1 nW | 10 μW | [2] |
| Ag/ZHO/GOQDs/ZHO/Pt | 0.08~0.3 | -0.01~-0.14 | 0.1 μW | 10 μW | [3] |
| Pt/CdSe-ZnS/Azurin/Au | 2.0 | -2.0 | 10 nW | 0.1 μW | [4] |
| Ag/Ga_2_O_3_/PbS/Pt | 0.12~0.26 | -0.05~-0.19 | 10 nW | 10 μW | [5] |
| Cu/PVA/MoS_2_ QDs/PVA/Cu | 2.0 | -2.5 | 6 μW | 2.5 mW | [6] |
| FTO/MoS_2_ QDs/Al | -2.0 | 2.0 | 8 μW | 20 mW | [7] |
| Pt/CdSe-CdS QDs/TaO_x_/Ta | 0.4 | -0.9 | 40 μW | 2.7 mW | [8] |
| ITO/PEDOT: PSS/MoS_2_ QDs & PVA/Al | 1 | -2 | 1 mW | 20 mW | [9] |
| Cu/HZO/GeS/Pt | 0.2 | -0.1 | 2 nW | 55 μW | [10] |


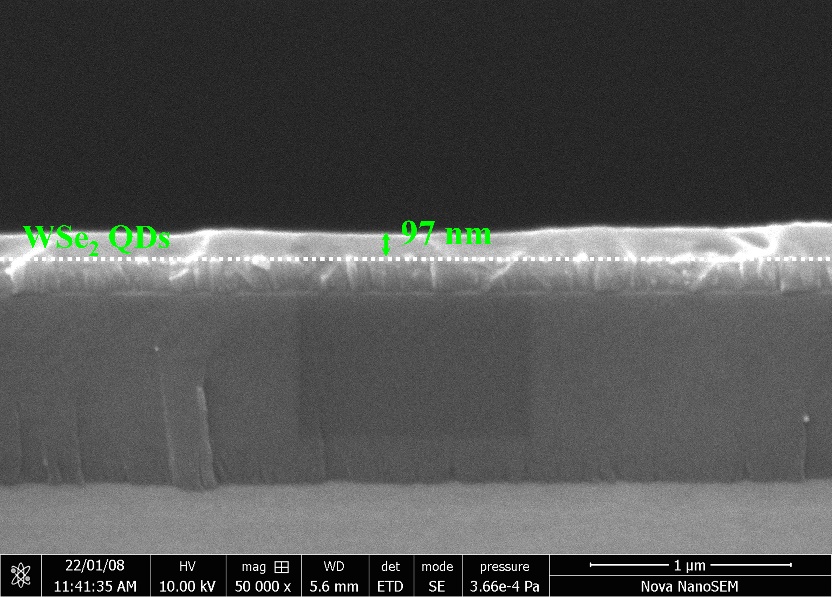


***Figure S1. The cross-sectional SEM image of the WSe_2_ QDs/LSMO/STO device.***


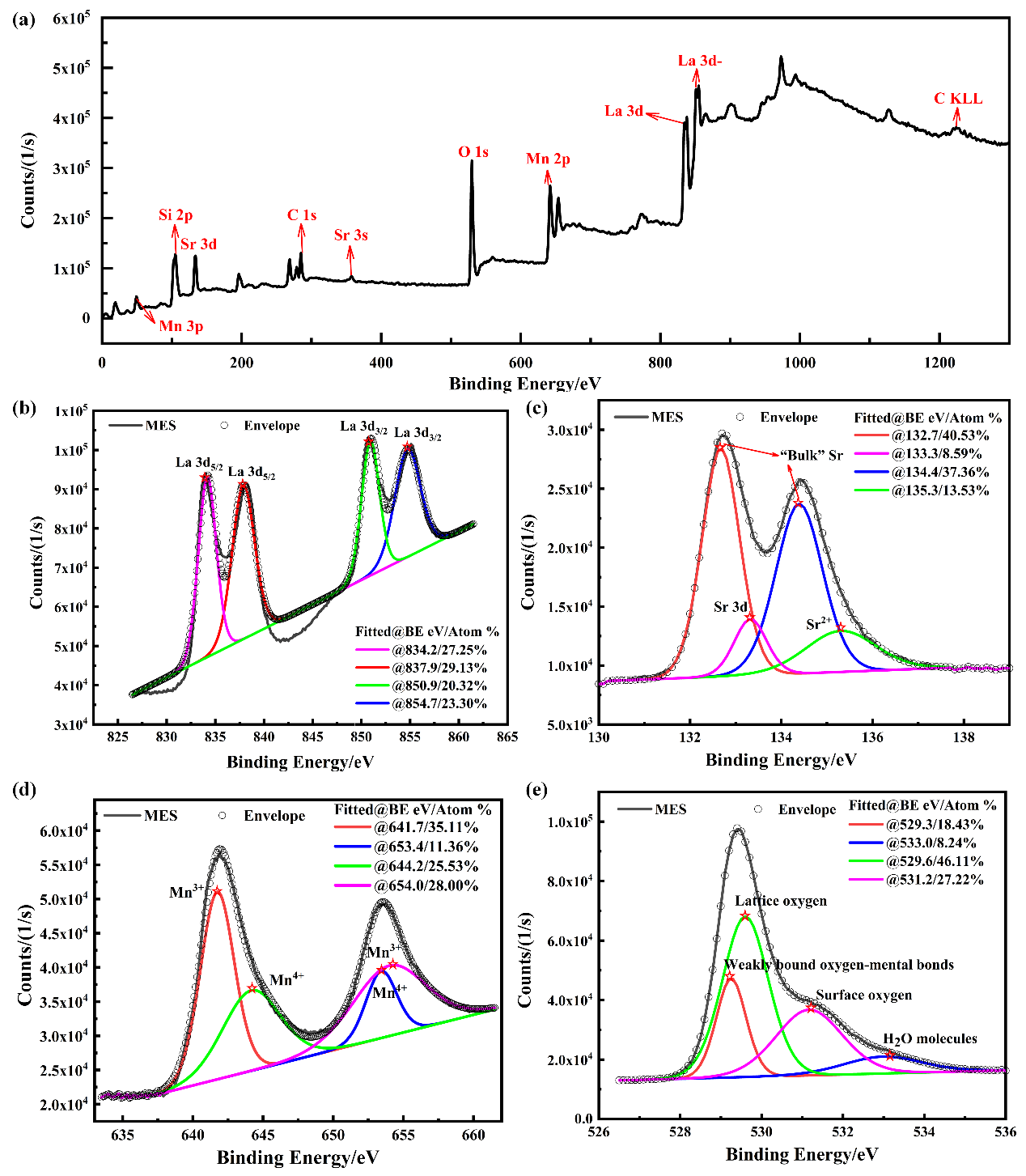


***Figure S2. XPS analysis result of the LSMO/STO device.*** *(a), wide spectra, (b)-(e) are the core spectra of La 3d, Sr 3d, Mn 2p and O 1s, respectively.*

Figure S2(a) shows the XPS wide spectra of the LSMO/STO device, from which the peak positions of the five elements C, O, La, Sr, Mn, and O can be clearly identified. According to the core spectra of La 3d in Figure S2(b), La 3d is divided into a La 3d_3/2_ double peak and a La 3d_5/2_ double peak. The peak position of La 3d_3/2_ appears at 850.9 eV and 854.7 eV, while the peak position of La 3d_3/2_ appears at 834.2 eV and 837.9 eV, confirming the presence of La^3+^ in LSMO film [11]. According to the core spectra of Sr 3d in Figure S2(c), the doublet at 132.7 eV and 134.4 eV can be attributed to “Bulk” Sr in LSMO [12]. The peak position of Sr 3d_5/2_ appears at 133.3 eV, corresponding to those in SrO-like secondary phases [13]. The peak located at 135.3 eV can be attributed to Sr^2+^in SrTiO_3_ [14]. The peaks located at 641.7 eV and 654.0 eV correspond to Mn^3+^ [11, 15], and the peaks located at 644.2 eV and 653.4 eV correspond to Mn^4+^ [16, 17](Figure S2(d)). As shown in Figure S2(e), the two peaks at the binding energies of ∼529.3 eV and ∼529.6 eV can be assigned to weakly bound oxygen-mental bounds in crystal lattice and lattice oxygen, respectively [18, 19]. The peak located at 531.2 eV corresponds to surface oxygen [20]. The peak located at 533.0 eV can be assigned to absorbed H_2_O molecules in LSMO/STO sample [19].





***Figure S3.*** ***XPS wide spectra of the WSe_2_ QDs/LSMO/STO device.***


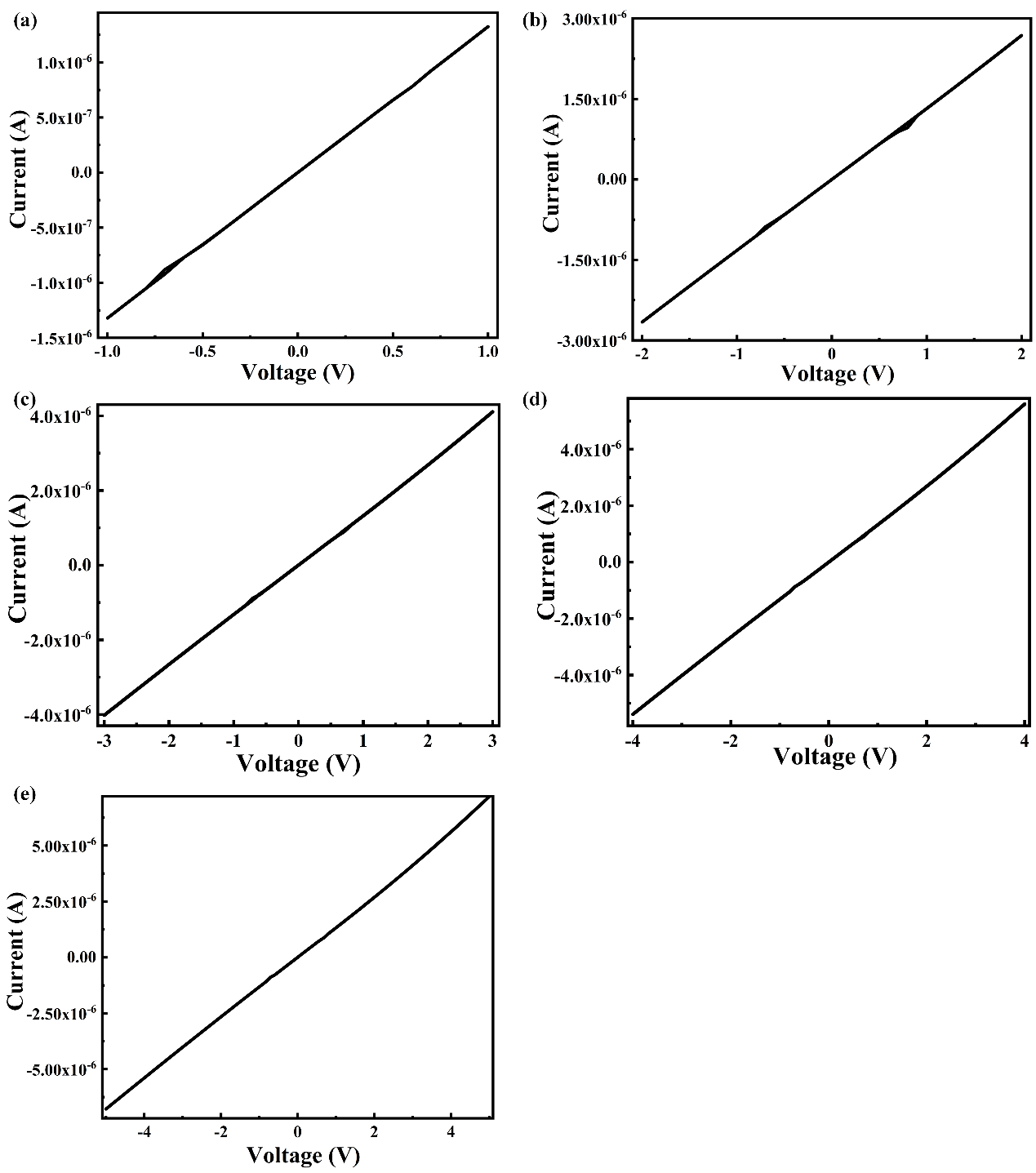


***Figure S4.*** *The I-V curves of the Ag/LSMO/STO device without spin-coated WSe_2_ QDs layer, the applied voltage sweep modes are* *0 V→1 V→0 V→-1 V→0 V (a), 0 V→2 V→0 V→-2 V→0 V (b), 0 V→3 V→0 V→-3 V→0 V (c), 0 V→4 V→0 V→-4 V→0 V (d) and 0 V→5 V→0 V→-5 V→0 V (e).*

**
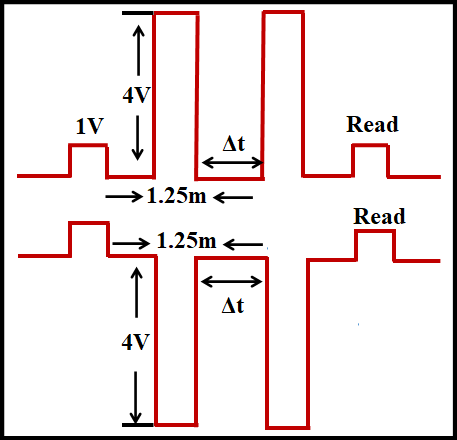
**

***Figure S5. Schematic diagram of the pulse waveforms applied to the device for PPF simulation.***

**
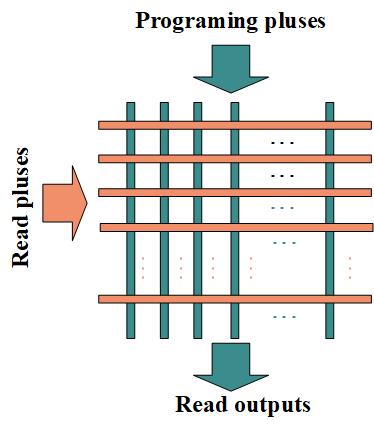
**

***Figure S6. The schematic diagram of the crossbar based on the WSe_2_ QDs device.*** *The intersections of the crossbar and the vertical bar in the crossbar all contain WSe_2_ QDs devices.*

**
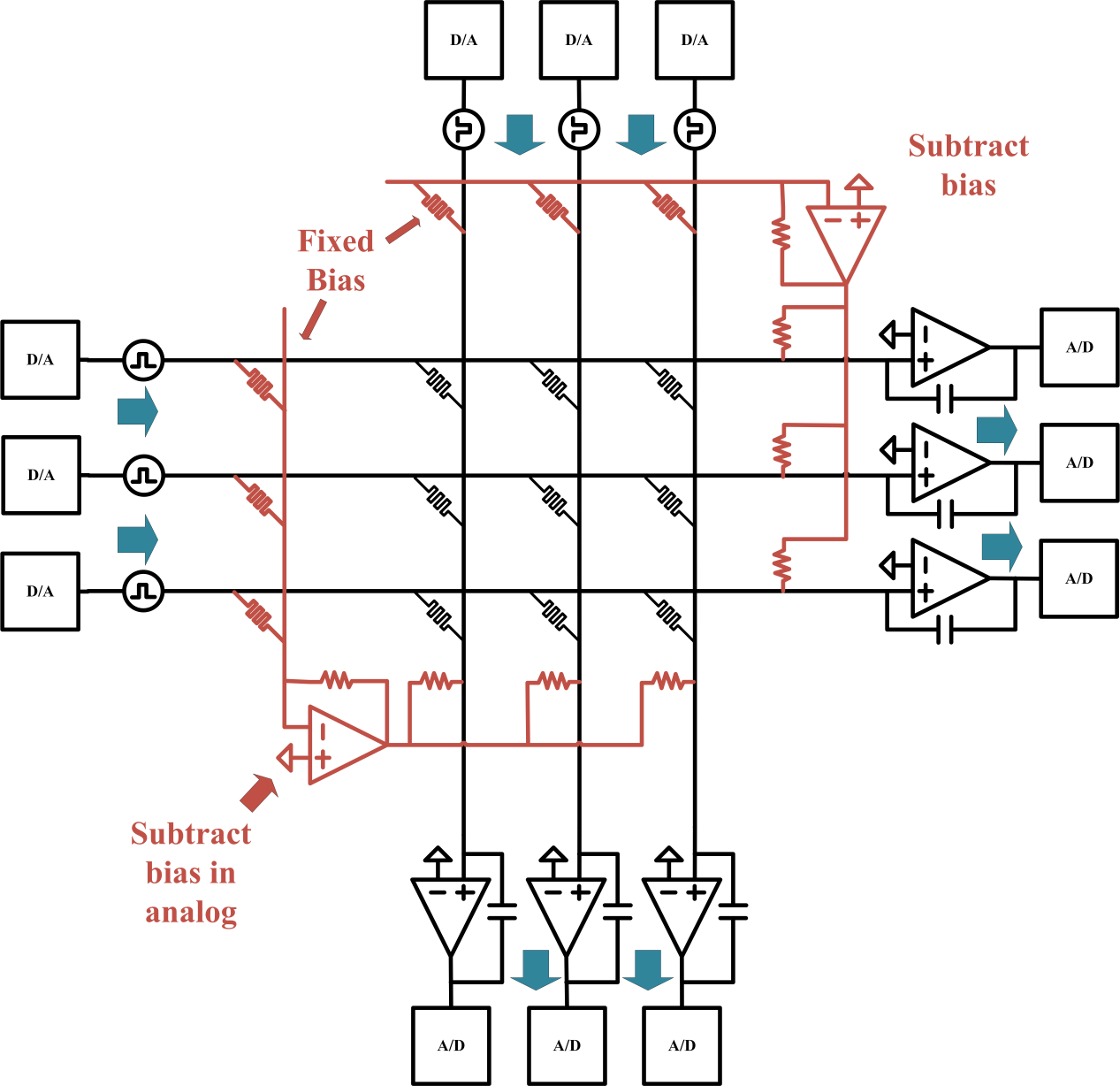
**

***Figure S7. The neural core [21].*** *To achieve negative weights, offset rows and columns are added to the Crossbar. An analog-to-digital converter is used to convert the output integrated current into a digital signal.*

**Computational** **details**

In our theoretical calculation, the density functional theory (DFT) method within Vienna *ab* initio simulation package (VASP) [22] were used. The Perdew-Burke-Ernzerh (PBE) [23] functional was used in the generalized gradient approximation (GGA) exchange-correlation potential. In order to avoid the interaction between periodically layers, the thickness of vacuum layer was set as 20 Å along the *z* direction. A 4 × 4 supercell (*a* = *b* = 13.308 Å, γ = 120°) in *ab* plane was used to investigate the atom defect properties of monolayer WSe_2_. The plane-wave cutoff energy was set as 500 eV and the *k*-point mesh in the Brillouin zone was chosen as 5×5×1. The atomic positions were optimized to converge until the force on each atom is 0.01 eV/Å, and the energy convergence is 10^-5^ eV in the electronic structure calculations.

The defect formation energy $\text{E}_{\text{f}}$ of uncharged defects is calculated by the following equation [24-26]:

$\text{E}_{\text{f}}\text{ = }\text{E}_{\text{defect}}^{\text{t}} \text{-}\text{ }\text{E}_{\text{pristine}}^{\text{t}} \text{- }\sum_{\text{i}} \text{∆}\text{N}_{\text{i}}\text{μ}_{\text{i}}$

where $\text{E}_{\text{defect}}^{\text{t}}$ represents the total energy of the defect systems, $\text{E}_{\text{pristine}}^{\text{t}}$ represents the total energy of the corresponding supercell without the defect, $\text{∆}\text{N}_{\text{i}}$ and $\text{μ}_{\text{i}}$ are the change in the number of atoms of species *i* induced by the formation of the defect and the chemical potential of species *i*, respectively. The chemical potentials $\text{μ}_{\text{W}}$ and $\text{μ}_{\text{Se}}$ are expressed as the limit values for the reduction of WSe_2_ to pure bcc W. The calculated defect formation energies for one Se-site defect and the composite defect models are listed in Table S2. The most preferred defect in energy is one Se*_d_* (1.84 eV). In the case of containing two Se*_d_*, the preferred defect configuration is Se*_d-opp_* (3.22 eV). Where a W*_d_* must be included, the preferred defect configuration is Se*_d-trans_* + W*_d_* (5.63 eV). According to the above study of defect formation energies, the achieved preferred defect models are Se*_d_*, Se*_d-opp_*, Se*_d-cis_*, Se*_d-trans_* and Se*_d-trans_* + W*_d_*.

***Table S2.*** ***The defect formation energies of defect configurations for WSe_2_.***

| Defect configuration | Defect formation energy (eV) |
| --- | --- |
| W*_d_* | 5.33 |
| Se*_d_* | 1.84 |
| Se*_d_* + W*_d_* | 5.42 |
| Se*_d-opp_* | 3.22 |
| Se*_d-cis_* | 3.71 |
| Se*_d-trans_* | 3.87 |
| Se*_d-opp_* + W*_d_* | 6.91 |
| Se*_d-cis_* + W*_d_* | 5.78 |
| Se*_d-trans_* + W*_d_* | 5.63 |

**Simulation process details**

In digits recognition, we encode the pixels of the digital image into different voltage pulses (as shown in Figure S8), and then input them into the crossbar. Synaptic weights are determined by a back-propagation algorithm and then directly mapped into conductance values. Based on Ohm's law and Kirchhoff's law, the crossbar containing the device can directly implement vector-matrix multiplication, which is also an advantage over traditional architectures. The input and output of the crossbar are driven by A/D and D/A converters with limited output range and bit precision, their ranges are listed in Table S3, and the largest of the final output values is the predicted number. The ranges in Table S3 are the ranges given by the algorithm, and values outside these ranges will be clipped. The bits here all include a sign bit. The row update range is proportional to the learning rate α, which is α = 0.1 in the simulation. The data used for the conductance parameter are the potentiation and depression behavior of the device, and the pulse conditions are: the negative voltage pulse part is -7→-0.2 V, the voltage change step is -0.2 V; the positive voltage pulse part is 0.2→7 V, the voltage change step is 0.2 V; the duration and interval of each pulse are both 41.5 μs.

**
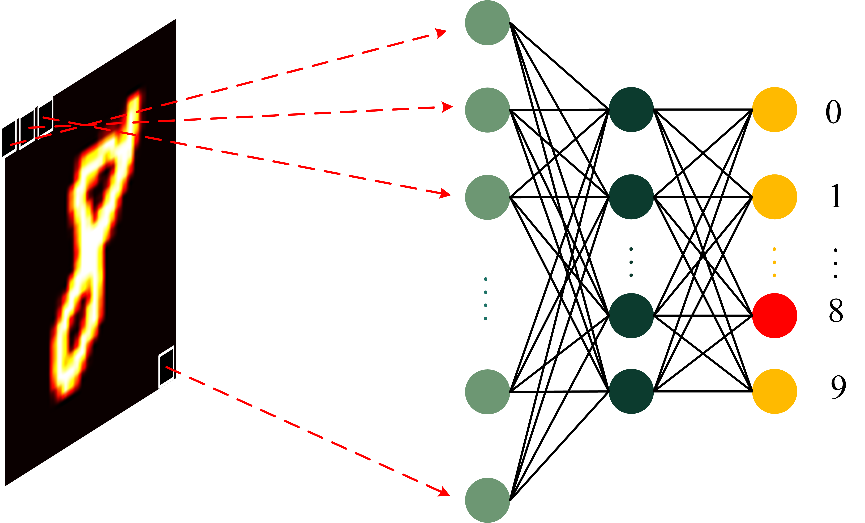
**

***Figure S8. Schematic diagram of digital image input.***

***Table S3. D/A and A/D attributes.***

|  | Range | Bits |
| --- | --- | --- |
| Row Input | -1 to 1 | 8 |
| Row Output | -6 to 6 | 8 |
| Col Input | -1 to 1 | 8 |
| Col Output | -4 to 4 | 8 |
| Row Update | -0.1α to 0.1α | 7 |
| Col Update | -1 to 1 | 5 |

**References**

[1] S. T. Han, Y. Zhou, B. Chen et al., “Hybrid flexible resistive random access memory-gated transistor for novel nonvolatile data storage,” *Small*, vol. 12, no. 3, pp. 390-396, 2016.

[2] Z. Wang, S. Joshi, S. E. Savel’ev et al., “Memristors with diffusive dynamics as synaptic emulators for neuromorphic computing,” *Nature materials*, vol. 16, no. 1, pp. 101-108, 2017.

[3] X. Yan, L. Zhang, Y. Yang et al., “Highly improved performance in Zr_0.5_Hf_0.5_O_2_ films inserted with graphene oxide quantum dots layer for resistive switching non-volatile memory,” *Journal of Materials Chemistry C*, vol. 5, no. 42, pp. 11046-11052, 2017.

[4] A. K. Yagati, S. U. Kim, T. Lee, J. Min, and J. W. Choi, “Recombinant azurin-CdSe/ZnS hybrid structures for nanoscale resistive random access memory device,” *Biosensors and Bioelectronics*, vol. 90, no., pp. 23-30, 2017.

[5] X. Yan, Y. Pei, H. Chen et al., “Self-assembled networked PbS distribution quantum dots for resistive switching and artificial synapse performance boost of memristors,” *Advanced materials*, vol. 31, no. 7, pp. 1805284, 2019.

[6] S. K. Ganeshan, V. Selamneni, and P. Sahatiya, “Water dissolvable MoS_2_ quantum dots/PVA film as an active material for destructible memristors,” *New Journal of Chemistry*, vol. 44, no. 28, pp. 11941-11948, 2020.

[7] A. Thomas, A. Resmi, A. Ganguly, and K. Jinesh, “Programmable electronic synapse and nonvolatile resistive switches using MoS_2_ quantum dots,” *Scientific Reports*, vol. 10, no. 1, pp. 1-10, 2020.

[8] J. Guo, S. Guo, X. Su et al., “Nonvolatile resistive switching memory device employing CdSe/CdS core/shell quantum dots as an electrode modification layer,” *ACS Applied Electronic Materials*, vol. 2, no. 3, pp. 827-837, 2020.

[9] M. Chen, G. Dong, X. Li et al., “Influence of MoS_2_ quantum dots size on the properties of memristor devices,” *Optik*, vol. 207, no., pp. 163776, 2020.

[10] Z. Zhu, Y. Pei, C. Gao, H. Wang, and X. Yan, “A Cu/HZO/GeS/Pt memristor for neuroinspired computing,” *Physica Status Solidi-Rapid Research Letters*, vol. 15, no. 10, pp. 2100072, 2021.

[11] Z. Duan, Y. Cui, X. Shi et al., “Facile fabrication of micro-patterned LSMO films with unchanged magnetic properties by photosensitive sol-gel method on LaAlO_3_ substrates,” *Ceramics International*, vol. 42, no. 12, pp. 14100-14106, 2016.

[12] M. P. de Jong, V. Dediu, C. Taliani, and W. R. Salaneck, “Electronic structure of La_0.7_Sr_0.3_MnO_3_ thin films for hybrid organic/inorganic spintronics applications,” *Journal of applied physics*, vol. 94, no. 11, pp. 7292-7296, 2003.

[13] H. Xie, H. Huang, N. Cao et al., “Effects of annealing on structure and composition of LSMO thin films,” *Physica B: Condensed Matter*, vol. 477, no., pp. 14-19, 2015.

[14] C. Q. Li, S. S. Yi, D. l. Chen et al., “Oxygen vacancy engineered SrTiO_3_ nanofibers for enhanced photocatalytic H_2_ production,” *Journal of Materials Chemistry A*, vol. 7, no. 30, pp. 17974-17980, 2019.

[15] C. S. Park, H. S. Lee, D. I. Shim et al., “The oxygen-deficiency-dependent Seebeck coefficient and electrical properties of mesoporous La_0.7_Sr_0.3_MnO_3-x_ films,” *Journal of materials chemistry A*, vol. 4, no. 12, pp. 4433-4439, 2016.

[16] H. B. Yang, “The structural and morphology of (La_0.6_Sr_0.4_)MnO_3_ thin films prepared by pulsed laser deposition,” *MATEC Web of Conferences*, vol. 44, no., pp. 02035, 2016.

[17] X. Chen, F. He, and S. Liu, “CuO/MnO_x_ composites obtained from Mn-MIL-100 precursors as efficient catalysts for the catalytic combustion of chlorobenzene,” *Reaction Kinetics, Mechanisms and Catalysis*, vol. 130, no. 2, pp. 1063-1076, 2020.

[18] H. F. Xiong, T. D. Cheng, X. G. Tang, J. Chen, and Q. X. Liu, “X-ray photoelectron spectroscopy of (La_0.7_Sr_0.3_)MnO_3_ thin films prepared by pulsed laser deposition,” *Advanced Materials Research*, vol. 284-286, no., pp. 2191-2197, 2011.

[19] K. Huang, X. Chu, W. Feng et al., “Catalytic behavior of electrospinning synthesized La_0.75_Sr_0.25_MnO_3_ nanofibers in the oxidation of CO and CH_4_,” *Chemical Engineering Journal*, vol. 244, no., pp. 27-32, 2014.

[20] J. Ling, K. Wang, Z. Wang, H. Huang, and G. Zhang, “Enhanced piezoelectric-induced catalysis of SrTiO_3_ nanocrystal with well-defined facets under ultrasonic vibration,” *Ultrason Sonochem*, vol. 61, no., pp. 104819, 2020.

[21] S. Agarwal, S. J. Plimpton, D. R. Hughart et al. “Resistive memory device requirements for a neural algorithm accelerator,” (*2016 International Joint Conference on Neural Networks (IJCNN),* *Vancouver, BC, Canada, July 2016)*, https://ieeexplore.ieee.org/stamp/stamp.jsp?tp=&arnumber=7727298.

[22] G. Kresse, and J. Furthmüller, “Efficient iterative schemes for ab initio total-energy calculations using a plane-wave basis set,” *Physical Review B*, vol. 54, no. 16, pp. 11169, 1996.

[23] J. P. Perdew, K. Burke, and M. Ernzerhof, “Generalized gradient approximation made simple,” *Physical review letters*, vol. 77, no. 18, pp. 3865, 1996.

[24] J. J. Yang, D. B. Strukov, and D. R. Stewart, “Memristive devices for computing,” *Nature nanotechnology*, vol. 8, no. 1, pp. 13-24, 2013.

[25] C. Du, W. Ma, T. Chang, P. Sheridan, and W. D. Lu, “Biorealistic implementation of synaptic functions with oxide memristors through internal ionic dynamics,” *Advanced Functional Materials*, vol. 25, no. 27, pp. 4290-4299, 2015.

[26] X. Yan, Q. Zhao, A. P. Chen et al., “Vacancy-induced synaptic behavior in 2D WS_2_ nanosheet-based memristor for low-power neuromorphic computing,” *Small*, vol. 15, no. 24, pp. 1901423, 2019.
